# Needs for information about lifestyle and rehabilitation in long-term young adult cancer survivors

**DOI:** 10.1007/s00520-021-06418-z

**Published:** 2021-08-01

**Authors:** Lene Thorsen, Synne-Kristin H. Bøhn, Hanne C. Lie, Sophie D. Fosså, Cecilie E. Kiserud

**Affiliations:** 1 National Advisory Unit On Late Effects After Cancer Treatment, Department of Oncology, Oslo University Hospital, Oslo, Norway; 2 Department of Clinical Service, Division of Cancer Medicine, Oslo University Hospital, Oslo, Norway; 3 Department of Behavioural Sciences in Medicine, Institute of Basic Medical Sciences, Faculty of Medicine, University of Oslo, Oslo, Norway; 4 Faculty of Medicine, University of Oslo, Oslo, Norway

**Keywords:** Long-term young adult cancer survivors, Information needs, Lifestyle advice, Physical activity/exercise advice, Dietary advice, Rehabilitation services

## Abstract

**Background:**

Healthy lifestyle and rehabilitation may mitigate late effects after cancer treatment, but knowledge about lifestyle and rehabilitation information needs among long-term young adult cancer survivors (YACSs) (≥ 5 years from diagnosis) is limited. The present study aimed to examine such information needs among long-term YACSs, and identify characteristics of those with needs.

**Material and methods:**

The Cancer Registry of Norway identified long-term YACSs diagnosed with breast cancer, colorectal cancer, non-Hodgkin lymphoma, leukemia, or malignant melanoma at the age of 19–39 years, between 1985 and 2009. Survivors were mailed a questionnaire, in which respondents reported their information needs on physical activity, diet, and rehabilitation services 5–30 years post-diagnosis. Descriptive statistics and logistic regression analyses were used to examine the prevalence of information needs and associated factors.

**Results:**

Of 1488 respondents (a response rate of 42%), 947 were included. Median age at diagnosis was 35 years (range 19–39) and median observation time since diagnosis was 14 years (range 5–30). In total, 41% reported information needs for information about physical activity, 45% about diet, and 47% about rehabilitation services. Information needs were associated with higher treatment intensity, increasing number of late effects, and an unhealthy lifestyle.

**Conclusion:**

A large proportion of long-term YACSs report information needs regarding lifestyle and/or rehabilitation more than a decade beyond treatment. Assessments of such information needs should become a part of long-term care of these cancer survivors.

**Supplementary Information:**

The online version contains supplementary material available at 10.1007/s00520-021-06418-z.

## Background

Young adults aged 19–39 years when diagnosed with cancer will often face unique challenges, such as disruptions to their education and career paths, and establishing relationships, form a family, and ensure financial stability, compared to those diagnosed in later adult age [1, 2]. Successful cancer treatment in young adulthood is typically associated with a long life expectancy, but also an increased risk of late effects due to the cancer and its treatment [3]. For example, research from the USA show that about 40% of long-term survivors of adolescent and young adult cancers have at least one severe or life-threatening late effect, including cardiovascular diseases, obesity, and abnormal pulmonary function by the age of 45 years [4].

A healthy lifestyle may reduce risks of late effects, potentially improving long-term health among cancer survivors [5]. In order to achieve a healthy lifestyle and to reach other health outcomes, cancer survivors may benefit from rehabilitation programs [6]. Such programs are typically delivered as inpatient multidisciplinary rehabilitation programs, outpatient single directed programs, or information emphasizing physical activity, nutrition, smoking dissertation, or physiological well-being. Studies show beneficial effects of several rehabilitation programs on lifestyle and physical and psychological health outcomes among cancer survivors [6, 7].

A large proportion of young adult cancer survivors (YACSs) report various information needs related to health outcomes and lifestyle during the first years after diagnosis [8] (see overview in the Supplementary File). According to Keegan and colleagues, 51% reported information needs about late effects, 32% about physical activity, and 40% about nutrition and diet among adolescent and YACSs (15–39 years of age at diagnosis) within 2 years after diagnosis [9]. The information needs later in the survivorship continuum are less studied. Among 160 adolescent and YACSs on average 12 years after various diagnoses, 70–80% reported information needs about late effects and follow-up, but their need for information about lifestyle and rehabilitation was not examined [10].

The current literature base includes studies examining information needs among YACSs in the first years after treatment [8, 9, 11, 12], but large-scale studies investigating information needs several years after treatment are lacking, as well as studies identifying subgroups of YACSs with needs for information on lifestyle and rehabilitation services specifically. By identifying information needs regarding lifestyle advice and rehabilitation programs among subgroups of YACSs, health care personnel are better prepared to deliver targeted information to those in need and help YACSs to make informed decisions about their lifestyle behavior and participation in rehabilitation programs.

The aims of this study were therefore to examine information needs about physical activity, diet, and rehabilitation services among long-term YACSs exclusively, and to identify demographic, medical, and lifestyle characteristics of those with such information needs. Due to long-term late effects impacting lifestyle and health, we hypothesize that the proportion of long-term YACSs who report information needs about lifestyle and rehabilitation will be at least as high as the proportion reported by YACSs in the first years after diagnosis in previous studies.

## Materials and methods

### Study participants

The current study is a sub-study of the nationwide, population-based NOR-CAYACS study [13]. Norwegian childhood, adolescent, and young adult cancer survivors (CAYACS) were identified through the Cancer Registry of Norway (CRN) and mailed a questionnaire-based health survey in 2015/2016.

Participants from the NOR-CAYACS study were included in this sub-study if ≥ 5 years had elapsed since a diagnosis of breast cancer (BC) (stages I–III), colorectal cancer (CRC), non-Hodgkin lymphoma (NHL), leukemia (LEUK), or malignant melanoma (MM) (localized treated with minimal surgery), between 1985 and 2009, during young adulthood (19–39 years of age). Relatively good prognosis and risk of late effects are reasons why these cancer diagnoses were chosen for inclusion. YACSs treated for other cancer types relevant for young adults, such as Hodgkin lymphoma, cervical cancer, and testicular cancer, were not included because these survivors were included in other concurrent studies at our department at the time of survey.

YACSs were excluded if more than one cancer diagnosis or distant metastases were registered in the CRN, if the participants reported to be on cancer treatment at the time of survey, if they reported recurrence of cancer, or if they did not respond to questions related to treatment and/or about information needs. Survivors after non-metastatic MM treated with minimal surgery served as a reference group for treatment intensity.

### Data sources and measurements

#### CRN data

Information on gender, date of birth, cancer diagnosis, and date of diagnosis were obtained from the CRN. This information was used to calculate age at diagnosis, age at survey, and time from diagnosis to survey.

#### Questionnaire data

In total, the questionnaire consisted of 302 items of which 162 items were compulsory. The topics covered were socio-demographic background, late effects, health care use and needs, information needs, work ability and financial burden, physical health, mental health, fatigue, lifestyle, health-related quality of life, and health literacy. The majority of the measures were covered by validated instruments [13–18].

Participants received the questionnaire by mail, together with study information, an informed consent form, and a pre-paid return envelope. Non-responders received one reminder after 5 months.

#### Outcome variables

Perceived information needs on lifestyle advice and rehabilitation services were assessed by three single questions: “Do you want advice on physical activity/exercise?”; “Do you want dietary advice?”; and “Do you want information about rehabilitation services?” The response categories for each question were “Yes,” “No, have no need,” and “No, have had need, but received enough information.” To identify characteristics of those with information needs, we chose to compare those who responded “Yes” with those who responded “No, have no need.”

#### Socio-demographic variables

We obtained information about marital status (living as a couple versus not), education (low level ≤ high school versus high level; i.e., college/university), and work situation (within work force/being a student versus not). Socio-economic status (SES) was assessed by combining marital status, education, and work situation. To be included in the high SES group, at least two of the three following conditions had to be fulfilled: living as a couple, college/university, and being within work force/being a student.

#### Treatment and late effects

Treatment was self-reported and categorized into (1) “minimal surgery restricted to localized MM” (surgical removal of the skin lesion only), (2) “surgery and/or radiotherapy only,” (3) “systemic treatment only,” and (4) “systemic treatment combined with surgery and/or radiotherapy.” Information on late effects was obtained by asking if the participants had experienced any of 16 listed late effects (hormonal changes, reduced fertility, cardiovascular diseases, lung problems, problems of dental health, problems with memory and concentration, problems with hearing, muscular cramps, peripheral neuropathic pain, numbness of hands/feet, sexual problems, osteoporosis, lymphedema, and radiation injuries). The total number of late effects was summarized for each participant and categorized into groups with 0, 1–2, 3–4, and ≥ 5 late effects. Chronic fatigue and psychological reactions were excluded from the list, since these conditions were measured by separate, validated instruments.

Fatigue was assessed by the Chalder Fatigue Questionnaire (FQ) [15]. FQ consists of 11 items (e.g., During the last month, “*Do you have problems with tiredness*?”; “*Do you have difficulty concentrating?*”) scored from 0 to 3, with increasing total score (0 to 33) implying higher levels of fatigue. Internal consistency (Cronbach’s alpha) for the population included in this analysis was 0.92 for fatigue scale. To identify chronic fatigue, scores of each item were dichotomized (0 = 0, 1 = 0, 2 = 1, 3 = 1) and chronic fatigue was defined by a dichotomized sum score ≥ 4 with ≥ 6 months duration [15].

Depressive symptoms were assessed using the Patient Health Questionnaire-9 (PHQ-9) [16]. PHQ-9 consists of 9 items (e.g., Over the last 2 weeks, how often have you been bothered by “*Little interest or pleasure in doing things*,” “*Feeling down, depressed, or hopeless*”) scored from 0 to 3, with increasing sum score (0 to 27) indicating higher level of depressive symptoms. Cronbach’s alpha for PHQ-9 was 0.88 in the present population. Anxiety symptoms were measured by the anxiety subscale of the Hospital Anxiety and Depression Scale (HADS-A) [18]. This subscale consists of 7 items (e.g., During the last week, can you describe how often you “*I feel tense or “wound up”*,” “*I get a sort of frightened feeling as if something awful is about to happen*,” “*Worrying thoughts go through my mind*”), scored from 0 to3, with increasing sum scores (0 to 21) indicating higher level of anxiety symptoms. Cronbach’s alpha for HADS-A was 0.83 in the present population.

#### Lifestyle variables

Physical activity/exercise was assessed by a modified version of the Godin Leisure Time Exercise Questionnaire [14]. Being physically inactive was defined as not meeting the guidelines of at least 150 min of moderate intensity, or 75 min of high intensity, or an equivalent combination of moderate and high intensity physical activity per week [19]. Being obese (defined as BMI (kg/m^2^) ≥ 30) was calculated from self-reported height and weight [20]. Being a current smoker was defined by responding “Yes, I smoke daily” to the question “Do you smoke?”.

### Statistical analyses

Continuous variables are presented by means, standard deviations (SD), medians, and ranges, and categorical variables by numbers and percentages. Three logistic regression analyses identified characteristics of YACSs with information needs on (1) physical activity, (2) diet, and (3) rehabilitation, compared to those without such needs. Statistically significant variables associated with each of the three types of needs in univariate analyses were included as explanatory variables in three separate multivariable logistic regression analyses (Tables 2, 3, and 4). Cancer diagnoses and depression symptoms were not included in the multivariable analyses due to high correlation with treatment group and chronic fatigue, respectively. Odds ratios (ORs) are presented with 95% confidence intervals (95% CI). Analyses were performed by SPSS 25.0 (SPSS, Chicago, IL). A *P* value ≤ 0.05 was considered statistically significant.

## Results

**Fig. 1:**
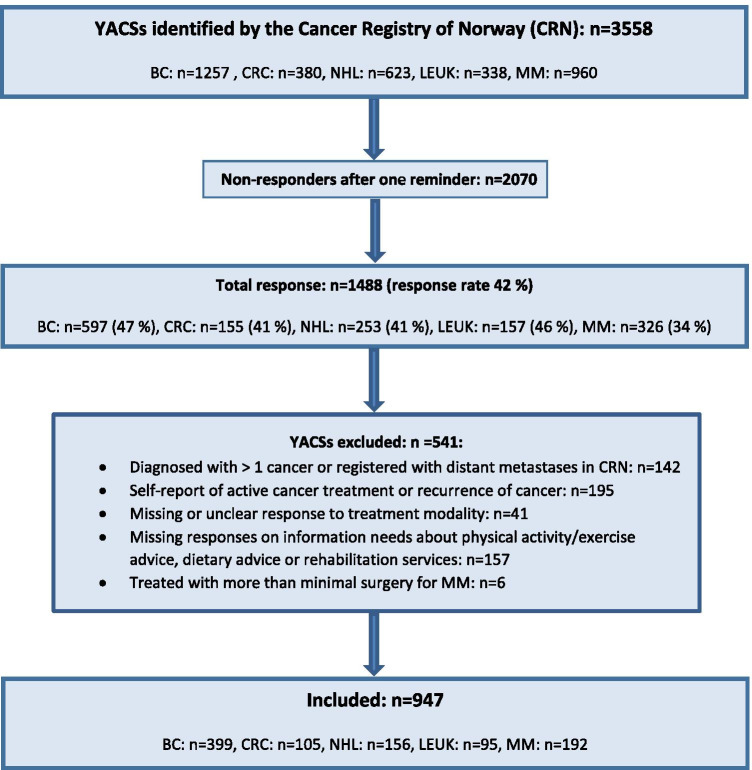
Flow chart for the current sub-study. BC breast cancer, CRC colorectal cancer, NHL non-Hodgkin lymphoma, LEUK leukemia, MM malignant melanoma

Of 3558 YACSs identified by the CRN and invited to participate, 1488 (42%) responded to the survey, of which 541 were excluded for the present analyses (Fig. 1). Among the 947 YACSs included, 74% were females, and 42% were diagnosed with BC (Table 1). Median age at diagnosis was 35 years (range 19–39), and median age at survey was 48 years (range 27–65). Median observation time since diagnosis was 14 years (range 5–30). Thirty-seven percent reported ≥ 3 late effects.

**Table 1:** Characteristics of participants (*n* = 947)

Socio-demographic variables	
Gender, *n* (%)	
Female	704 (74)
Male	243 (26)
Age at survey (years) (mean (SD)/median (range))	49 (7.6)/48 (27–65)
Age categories, *n* (%)	
< 50 years	558 (59)
≥ 50 years	389 (41)
Marital status, *n* (%)	
Living as a couple	752 (80)
Not living as a couple	190 (20)
Education, *n* (%)	
High level (college/university)	557 (59)
Low level (≤ high school)	382 (41)
Work situation, *n* (%)	
Within work force	786 (84)
Not within work force	153 (16)
Socio-economic status^a^, *n* (%)	
High	770 (82)
Low	169 (18)
Cancer-related variables and late effects	
Age at diagnosis (years) (mean (SD)/median (range))	33 (5.4)/35 (19–39)
Years since diagnosis (mean (SD)/median (range))	15 (6.7)/14 (5–30)
Diagnoses, *n* (%)	
Breast cancer	399 (42)
Colorectal cancer	105 (11)
Non-Hodgkin lymphoma	156 (17)
Leukemia	95 (10)
Malignant melanoma	192 (20)
Treatment modality, *n* (%)	
Minimal surgery	192 (20)
Surgery and/or radiotherapy only	143 (15)
Systemic treatment only	133 (14)
Systemic treatment combined with surgery and/or radiotherapy	479 (51)
Number of late effects^b^	
0	360 (38)
1–2	241 (26)
3–4	174 (19)
> 5	164 (18)
Chronic fatigue, *n* (%)	
No	694 (74)
Yes	241 (26)
HADS-A^c^ score (mean (SD)/median (range))	4.9 (3.8)/4 (0–21)
PHQ-9^d^ score (total) (mean (SD)/median (range))	5.6 (4.9)/4 (0–27)
Lifestyle variables, ***n*** (%)	
Inactive^e^	
No	506 (55)
Yes	413 (45)
Obese (BMI ≥ 30) (kg/m^2^)	
No	775 (83)
Yes	154 (17)
Current daily smoker	
No	831 (88)
Yes	113 (12)

*SD* standard deviation, *BMI* body mass index

Numbers may not add up to 947 because of missing data and percentages may not add up to 100 because of rounding

^**a**^**Socio-economic status** is calculated by merging marital status (living as a couple versus not) and education (high level versus low level and work situation (within work force/being a student versus not). If the participants lived as a couple, had a high level of education, and were within work force/being a student, or if they fulfilled two of these, they were categorized into a high socio-economic status group. If the participants were not living as a couple, had a low level of education, and were not within work force/not being a student, or if they fulfilled two of these, they were categorized into the low socio-economic status group

^**b**^**Number of late effects** included the following: hormonal changes, reduced fertility, cardiovascular diseases, lung problems, problems of dental health, problems with memory and concentration, problems with hearing, muscular cramps, nerve pains, numbness of hands/feet, sexual problems, osteoporosis, lymphedema, and radiation injuries (no/yes) summarized for each participant and categorized into participants with 0, 1–2, 3–4, and ≥ 5. Chronic fatigue and psychological reactions were excluded from the list of late effects

^**c**^**HADS-A** = Hospital Anxiety and Depression Scale, Anxiety subscale. Increasing scores imply worse symptoms

^**d**^**PHQ-9** = The Patient Health Questionnaire-9. Increasing scores imply worse symptoms. Not included in multivariate analyses, because of high correlation to fatigue

^**e**^**Inactive** defined as not meeting the PA guidelines of at least 150 min of moderate intensity, 75 min high intensity, or an equivalent combination of moderate and high intensity PA per week

### Information needs

**Fig. 2:**
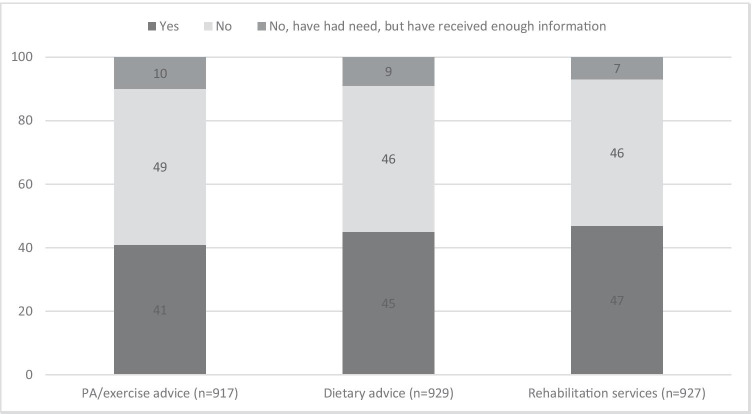
Proportion of YACSs with information needs on physical activity, diet, and rehabilitation

Among all participants, 41% reported information needs on physical activity, 45% on diet, and 47% on rehabilitation services (Fig. 2). Ten percent or less reported that they had had information needs, but had received enough information in these areas. Twenty-seven percent had information needs on physical activity, diet, and rehabilitation services, 15% had two information needs, 19% had one information need, and 39% had no information need.

### Characteristics of YACSs with information needs

#### Lifestyle

Multivariable logistic regression analysis showed that YACSs who had received systemic treatment in combination with surgery and/or radiotherapy were more likely to report needs for information about physical activity and diet compared to our reference group of MM survivors (Tables 2 and 3). YACSs with chronic fatigue, who had increasing symptoms of anxiety, who were physically inactive, or who were obese were also more likely to have a need for information on lifestyle than those without these characteristics (Tables 2 and 3).

**Table 2:** Proportion of YACSs with information needs on physical activity and characteristics of YACSs with this information need

Physical activity/exercise advice (*n* = 823)			Univariate analyses			Multivariable analyses			
Variables	Yes (*n* = 373)	No, need (*n* = 450)	OR	95% CI	*P* value	aOR	95% CI		*P* value
Sex, *n* (%)									
Female	275 (45)	338 (55)	1.00						
Male	98 (47)	112 (53)	1.08	0.79–1.47	0.650				
Age at survey 2 categories, *n* (%)									
≥ 50	132 (39)	207 (61)	1.00			1.0			
< 50	241 (50)	243 (50)	1.56	1.17–2.06	**0.002**	1.20		.79–1.81	.390
Socio-economic status^a^, *n* (%)									
High	291 (44)	377 (56)	1.00			1.0			
Low	80 (54)	69 (46)	1.50	1.05–2.15	**0.025**	0.96		0.65–1.53	0.983
**Cancer-related variables and late effects**									
Years since diagnosis									
5–10 years	145 (54)	126 (46)	1.00			1.00			
11–20 years	154 (43)	203 (57)	0.66	0.48–0.91	**0.010**	0.77		0.51–1.16	.215
21–30 years	74 (38)	121 (62)	0.53	0.37–0.77	**0.001**	0.76		0.44–1.32	.333
Diagnoses, *n* (%)									
Malignant melanoma	52 (30)	123 (70)	1.00						
Breast cancer	172 (50)	172 (50)	2.37	1.61–3.48	** < .001**				
Colorectal cancer	43 (47)	49 (53)	2.08	1.23–3.50	**0.006**				
Non-Hodgkin lymphoma	66 (50)	65 (50)	2.40	1.50–3.85	** < .001**				
Leukemia	40 (49)	41 (51)	2.31	1.34–3.97	**0.003**				
Treatment modality, *n* (%)									
Minimal surgery	52 (30)	123 (70)	1.0			1.0			
Surgery and/or radiotherapy	50 (40)	76 (60)	1.56	0.96–2.52	**0.072**	1.74		0.99–3.05	0.056
Systemic treatment only	54 (48)	59 (52)	2.17	1.33–3.54	**0.002**	1.81		0.99–3.30	0.053
Systemic treatment with surgery and/or radiotherapy	217 (53)	192 (47)	2.67	1.83–3.90	** < .001**	**2.18**		**1.29–3.68**	**0.004**
Number of late effects^b^, *n* (%)									
0	111 (34)	217 (66)	1.0			1.0			
1–2	97 (46)	116 (55)	1.64	1.15–2.33	**0.006**	1.03		0.66–1.61	0.885
3–4	84 (58)	60 (42)	2.74	1.83–4.09	** < .001**	1.55		0.94–2.55	0.086
≥ 5	77 (58)	55 (42)	2.74	1.81–4.14	** < .001**	1.23		0.71–2.11	0.464
Chronic fatigue, *n* (%)									
No	235 (38)	381 (62)	1.0			**1.0**			
Yes	136 (68)	63 (32)	3.5	2.49–4.92	** < .001**	**2.14**		**1.45–3.17**	** < .001**
HADS-A^c^ score, mean (SD)	5.8 (4.0)	3.9 (3.4)	1.15	1.10–1.19	** < .001**	**1.10**		**1.06–1.16**	** < .001**
PHQ-9^d^ score, mean (SD)	7.2 (5.3)	4.1 (4.0)	1.16	1.12–1.20	** < .001**				
**Lifestyle variables, *n* (%)**									
Inactive^e^									
No	174 (40)	264 (60)	1.0			**1.0**			
Yes	190 (52)	173 (48)	1.67	1.26–2.21	** < .001**	**1.59**		**1.16–2.18**	**.004**
BMI ≥ 30 kg/m^2^									
No	283 (42)	392 (58)	1.0			**1.0**			
Yes	78 (59)	54 (41)	2.00	1.37–2.92	** < .001**	**1.86**		**1.22–2.84**	**.004**

*OR* odds ratio, *aOR* adjusted odds ratio, *SD* standard deviation, *BMI* body mass index

Numbers may not add up to 823 because of missing data and percentages may not add up to 100 because of rounding

^**a**^**Socio-economic status** is calculated by merging marital status (living as a couple versus not) and education (high level versus low level and work situation (within work force/being a student versus not). If the participants lived as a couple, had a high level of education, and were within work force/being a student, or if they fulfilled two of these, they were categorized into a high socio-economic status group. If the participants were not living as a couple, had a low level of education, and were not within work force/not being a student, or if they fulfilled two of these, they were categorized into the low socio-economic status group

^**b**^**Number of late effects** included the following: hormonal changes, reduced fertility, cardiovascular diseases, lung problems, problems of dental health, problems with memory and concentration, problems with hearing, muscular cramps, nerve pains, numbness of hands/feet, sexual problems, osteoporosis, lymphedema, and radiation injuries (no/yes) summarized for each participant and categorized into participants with 0, 1–2, 3–4, and ≥ 5. Chronic fatigue and psychological reactions were excluded from the list of late effects

^**c**^**HADS-A** = Hospital Anxiety and Depression Scale, Anxiety subscale. Increasing scores imply worse symptoms

^**d**^**PHQ-9** = The Patient Health Questionnaire-9. Increasing scores imply worse symptoms. Not included in multivariate analyses, because of high correlation to fatigue

^**e**^**Inactive** defined as not meeting the PA guidelines of at least 150 min of moderate intensity, 75 min high intensity, or an equivalent combination of moderate and high intensity PA per week

**Bold**: *P* value < .05

**Table 3:** Proportion of YACSs with information needs on diet and characteristics of YACSs with this information need

Dietary advice (*n* = 843)				Univariate analyses			Multivariable analyses		
Variables	Yes (*n* = 414)		No, need (*n* = 429)	OR	95% CI	*P* value	aOR	95% CI	*P* value
Sex, *n* (%)									
Female	300 (48)		330 (52)	1.00					
Male	114 (54)		99 (47)	1.27	0.93–1.73	0.137			
Age at survey 2 categories, *n* (%)									
≥ 50	146 (43)		197 (57)	1.00			1.0		
< 50	268 (54)		232 (46)	1.56	1.18–2.06	**0.002**	1.20	.81–1.79	
Socio-economic status^a^, *n* (%)									
High	325 (48)		358 (52)	1.00					
Low	84 (55)		68 (45)	1.36	0.96–1.94	0.087			
**Cancer-related variables and late effects**									
Years since diagnosis									
5–10 years	161 (58)	118 (42)		1.00			1.00		
11–20 years	169 (46)	199 (54)		0.62	0.46–0.85	**0.003**	0.73	0.49–1.09	0.126
21–30 years	84 (43)	112 (57)		0.55	0.38–0.80	**0.001**	0.77	0.45–1.30	0.328
Diagnoses, *n* (%)									
Malignant melanoma	64 (36)		115 (64)	1.00					
Breast cancer	183 (52)		168 (48)	1.96	1.35–2.84	** < .001**			
Colorectal cancer	51 (55)		41 (45)	2.24	1.34–3.73	**0.002**			
Non-Hodgkin lymphoma	70 (52)		65 (48)	1.94	1.23–3.05	**0.004**			
Leukemia	46 (54)		40 (46)	2.07	1.23–3.48	**0.006**			
Treatment modality, *n* (%)									
Minimal surgery	64 (36)		115 (64)	1.0			1.0		
Surgery and/or radiotherapy	55 (44)		69 (56)	1.43	0.90–2.29	**0.132**	**1.73**	**1.0–2.97**	**0.049**
Systemic treatment only	62 (52)		57 (48)	1.95	1.22–3.13	**0.005**	**1.84**	**1.04–3.27**	**0.036**
Systemic treatment with surgery and/or radiotherapy	233 (55)		188 (45)	2.23	1.55–3.20	** < .001**	**1.94**	**1.18–3.20**	**0.009**
Number of late effects^b^, *n* (%)									
0	130 (40)		196 (60)	1.0			1.0		
1–2	105 (48)		114 (52)	1.39	0.98–1.96	**0.063**	0.87	0.56–1.33	0.508
3–4	84 (56)		66 (44)	1.92	1.30–2.84	**0.001**	1.11	0.68–1.81	0.669
≥ 5	89 (64)		51 (36)	2.63	1.75–3.96	** < .001**	1.20	0.71–2.03	0.502
Chronic fatigue									
No	264 (42)		361 (58)	1.0			**1.0**		
Yes	145 (70)		62 (30)	3.20	2.28–4.48	** < .001**	**2.09**	**1.42–3.08**	** < .001**
HADS-A^c^ score	5.7 (3.9)		3.9 (3.5)	1.14	1.10–1.18	** < .001**	**1.10**	**1.05–1.15**	** < .001**
PHQ-9^d^ score	7.0 (5.2)		4.1 (4.1)	1.14	1.11–1.18	** < .001**			
**Lifestyle variables, *n* (%)**									
Inactive^e^									
No	202 (45)		249 (55)	1.0			**1.0**		
Yes	203 (55)		167 (45)	1.50	1.14–1.98	**0.004**	**1.41**	**1.04–1.92**	**.027**
BMI ≥ 30 kg/m^2^									
No	321 (46)		372 (54)	1.0			**1.0**		
Yes	82 (61)		52 (39)	1.83	1.25–2.67	**0.002**	**1.69**	**1.11–2.57**	**.014**

*OR* odds ratio, *aOR* adjusted odds ratio, *SD* standard deviation, *BMI* body mass index

Numbers may not add up to 843 because of missing data and percentages may not add up to 100 because of rounding

^**a**^**Socio-economic status** is calculated by merging marital status (living as a couple versus not) and education (high level versus low level and work situation (within work force/being a student versus not). If the participants lived as a couple, had a high level of education, and were within work force/being a student, or if they fulfilled two of these, they were categorized into a high socio-economic status group. If the participants were not living as a couple, had a low level of education, and were not within work force/not being a student, or if they fulfilled two of these, they were categorized into the low socio-economic status group

^**b**^**Number of late effects** included the following: hormonal changes, reduced fertility, cardiovascular diseases, lung problems, problems of dental health, problems with memory and concentration, problems with hearing, muscular cramps, nerve pains, numbness of hands/feet, sexual problems, osteoporosis, lymphedema, and radiation injuries (no/yes) summarized for each participant and categorized into participants with 0, 1–2, 3–4, and ≥ 5. Chronic fatigue and psychological reactions were excluded from the list of late effects

^**c**^**HADS-A** = Hospital Anxiety and Depression Scale, Anxiety subscale. Increasing scores imply worse symptoms

^**d**^**PHQ-9** = The Patient Health Questionnaire-9. Increasing scores imply worse symptoms. Not included in multivariate analyses, because of high correlation to fatigue

^**e**^**Inactive** defined as not meeting the PA guidelines of at least 150 min of moderate intensity, 75 min high intensity, or an equivalent combination of moderate and high intensity PA per week

**Bold**: *P* value < .05

#### Rehabilitation

Multivariable logistic regression analysis showed that YACSs who had received more than minimal surgery for MM were more likely to have a need for information about rehabilitation services (Table 4). Those who reported ≥ 3 late effects were also more likely to have need for information on rehabilitation services than those reporting no late effects (Table 4). YACSs who reported chronic fatigue, who had increasing symptoms of anxiety, or who were obese were also more likely to want such information compared to those without these characteristics (Table 4).

**Table 4:** Proportion of YACSs of information needs on rehabilitation services and characteristics of YACSs with this information need

Information needs of available rehabilitation services (*n* = 862)			Univariate analyses				Multivariable analyses			
Variables	Yes (*n* = 437)	No, need (*n* = 425)	OR	95% CI		*P* value	aOR	95% CI		*P* value
Sex, *n* (%)										
Female	325 (51)	312 (49)	1.0							
Male	112 (50)	113 (50)	0.95	0.70–1.29		0.749				
Age at survey 2 categories, *n* (%)							1.0			
≥ 50	157 (45)	194 (55)	1.0				1.05		.68–1.61	.822
< 50	280 (55)	231 (45)	1.50	1.14–1.97		**0.004**				
Socio-economic status^a^, *n* (%)										
High	338 (48)	363 (52)	1.0				1.0			
Low	98 (63)	57 (37)	1.85	1.29–2.64		**0.001**	1.35		0.85–2.14	0.209
**Cancer-related variables and late effects**										
Years since diagnosis										
5–10 years	166 (58)	121 (42)	1.00				1.00			
11–20 years	187 (50)	184 (50)	0.62		0.46–0.85	**0.003**	0.96		0.63–1.46	0.842
21–30 years	84 (41)	120 (59)	0.55		0.38–0.80	**0.001**	0.73		0.41–1.29	0.280
Diagnoses, *n* (%)										
Malignant melanoma	41 (23)	140 (77)	1.00							
Breast cancer	213 (61)	137 (39)	5.31	3.53–7.99		** < .001**				
Colorectal cancer	52 (52)	49 (49)	3.62	2.15–6.11		** < .001**				
Non-Hodgkin lymphoma	86 (59)	60 (41)	4.89	3.03–7.91		** < .001**				
Leukemia	45 (54)	39 (46)	3.94	2.27–6.84		** < .001**				
Treatment modality, *n* (%)										
Minimal surgery	41 (23)	140 (77)	1.0				1.0			
Surgery and/or radiotherapy	57 (43)	75 (57)	2.60	1.59–4.24		** < .001**	2.50		1.40–4.47	**0.002**
Systemic treatment only	69 (57)	52 (43)	4.53	2.75–7.48		** < .001**	3.45		1.85–6.42	** < .001**
Systemic treatment with surgery and/or radiotherapy	270 (63)	158 (37)	5.84	3.91–8.70		** < .001**	3.63		2.10–6.27	** < .001**
Number of late effects^b^, *n* (%)										
0	110 (32)	231 (68)	1.0				1.0			
1–2	118 (53)	104 (47)	2.38	1.68–3.37		** < .001**	1.35		0.87–2.09	0.186
3–4	93 (60)	62 (40)	3.15	2.13–4.67		** < .001**	1.69		1.03–2.77	**0.037**
≥ 5	113 (81)	26 (19)	9.13	5.63–14.79		** < .001**	3.85		2.12–6.97	** < .001**
Chronic fatigue										
No	265 (42)	370 (58)	1.0				1.0			
Yes	167 (77)	51 (23)	4.57	3.22–6.49		** < .001**	2.33		1.53–3.53	** < .001**
HADS-A^c^ score, mean (SD)	5.9 (4.1)	3.7 (3.1)	1.18	1.14–1.23		** < .001**	1.15		1.09–1.20	** < .001**
PHQ-9 score^d^, mean (SD)	7.5 (5.2)	3.5 (3.4)	1.25	1.20–1.30		** < .001**				
**Lifestyle variables, *n* (%)**										
Inactive^e^										
No	209 (46)	243 (54)	1.0				1.0			
Yes	216 (56)	170 (44)	1.48	1.12–1.94		**0.005**	1.38		.99–1.90	0.055
Obese (BMI ≥ 30 kg/m^2^)										
No	347 (49)	363 (51)	1.0				1.0			
Yes	82 (59)	56 (41)	1.53	1.06–2.22		**0.024**	1.58		1.01–2.46	**.045**

*OR* odds ratio, *aOR* adjusted odds ratio, *SD* standard deviation, *BMI* body mass index

Numbers may not add up to 862 because of missing values and percentages may not add up to 100 because of rounding

^**a**^**Socio-economic status** is calculated by merging marital status (living as a couple versus not) and education (high level versus low level and work situation (within work force/being a student versus not). If the participants lived as a couple, had a high level of education, and were within work force/being a student, or if they fulfilled two of these, they were categorized into a high socio-economic status group. If the participants were not living as a couple, had a low level of education, and were not within work force/not being a student, or if they fulfilled two of these, they were categorized into the low socio-economic status group

^**b**^**Number of late effects** included the following: hormonal changes, reduced fertility, cardiovascular diseases, lung problems, problems of dental health, problems with memory and concentration, problems with hearing, muscular cramps, nerve pains, numbness of hands/feet, sexual problems, osteoporosis, lymphedema, and radiation injuries (no/yes) summarized for each participant and categorized into participants with 0, 1–2, 3–4, and ≥ 5. Chronic fatigue and psychological reactions were excluded from the list of late effects

^**c**^**HADS-A** = Hospital Anxiety and Depression Scale, Anxiety subscale. Increasing scores imply worse symptoms

^**d**^**PHQ-9** = The Patient Health Questionnaire-9. Increasing scores imply worse symptoms. Not included in multivariate analyses, because of high correlation to fatigue

^**e**^**Inactive** defined as not meeting the PA guidelines of at least 150 min of moderate intensity, 75 min high intensity, or an equivalent combination of moderate and high intensity PA per week

**Bold**: *P* value < .05

## Discussion

### Main findings

This large population-based study shows that a significant proportion of long-term YACSs have unmet information needs regarding lifestyle and rehabilitation services more than a decade beyond diagnosis and treatment. Survivors who have received treatments beyond minimal surgery, who have multiple late effects including chronic fatigue and mental distress, and who are physically inactive or obese also have higher information needs than those without these characteristics according to our results.

Our study expand knowledge about information needs in long-term YACSs, an understudied population with regard to survivorship compared to other age groups of cancer survivors [21]. To our knowledge, only one study has previously addressed information needs in long-term YACSs (> 5 years post-diagnosis). Christen et al. found that > 70% of long-term YACSs had information needs on late effects and follow-up; however, information needs on lifestyle and rehabilitation were not examined [10]. Zebrack found that more than half of 879 YACSs treated for different types of cancers on average 4.7 years post-diagnosis had unmet information need about diet and exercise [12], while McCarthy et al. showed that a third of AYA survivors within 2 years from diagnosis reported unmet information needs on diet and about staying physically fit [11]. Our study adds that these matters remain important for YACSs many years after diagnosis and treatment.

Keegan et al. suggested that information needs may increase throughout the post-treatment continuum [9]. In our study sample, needs for information about lifestyle and rehabilitation were similar among YACSs > 10 years from diagnosis compared to YACSs 5–10 years from diagnosis. This may indicate that YACSs, possibly due to long-term late effects, are unsure of what they should do to have a healthy lifestyle even several years after diagnosis.

More than 60% of YACSs who had received systemic treatment with surgery and/or radiotherapy needed information on rehabilitation services, compared to less than 25% of those who had undergone minimal surgery. In contrast, Keegan et al. did not demonstrate difference between treatment groups in relation to unmet information needs among adolescent and YACSs in a median of 11 months from diagnosis [9].

As expected, participants who reported five or more late effects were almost four times more likely to report need for information about rehabilitation services than those without late effects, but were not more likely to report need for information on physical activity and diet. This may indicate that YACSs with high burden of late effects call for multidisciplinary services, rather than advice on single items such as physical activity and nutrition. On the other hand, participants with chronic fatigue were twice as likely to report a need for all three types of information compared to those without chronic fatigue. We are not aware of other studies that have investigated the association between late effects and unmet information needs among long-term YACSs; however, Zebrack found that survivors who reported excellent/very good health status were less likely to report unmet need for information on exercise, diet, and programs on camps/retreats than those who reported fair and poor health status [12]. Moreover, Keegan et al. observed that survivors with health or emotional problems that interfere with their activities or survivors with three or more symptoms were more likely to report unmet information needs [9]. Previous findings from the NOR-CAYACS study show that less than half of the participants were physically active and less than 10% fulfilled the public diet recommendation regarding 5 a day (five fruit or vegetables per day) [22]. The reason why a large proportion of YACSs have an unhealthy lifestyle might be lack of specific knowledge of what kind of activities they should perform and what they should eat. We also found that the need for information about physical activity and diet was significantly higher among the physically inactive and obese, suggesting that these subgroups are motivated for lifestyle change.

In addition to YACSs, health care personnel must be informed about late effects and management of these, including lifestyle and rehabilitation services, to enable YACSs to make informed decisions about their lifestyle behavior and participation in rehabilitation programs. As such, the results of our study may help health care personnel to identify subgroups of YACSs in need of lifestyle advice and rehabilitation services. In general, a better organization of survivorship care with more information to the survivors and improved better communication between health care personnel in hospitals, general practitioners, and rehabilitation institutions might have a positive impact on the level of knowledge and thus potentially the long-term health of the cancer survivors.

### Limitations and strengths

A limitation of the study is the modest, yet increasingly common, response rate. We cannot rule out that non-responders have other information needs than the responders. However, previous analyses based on information of the whole population provided by the CRN found low risk of non-response bias in the NOR-CAYACS cohort [13]. Our findings suggest several associations between information needs and health outcomes, but the cross-sectional design prevents us from exploring causal relationships. Measuring information needs with a single-item question provides a general picture on the frequency of YACSs who have need for information, but do not provide detailed information on what kind of physical activity and exercise and/or dietary advice they are in need of (general or specific advice, home-based or supervised programs, individual or in groups, etc.), what type of rehabilitation the participants want (in- or out-patients program, which components, duration, etc.), how the information should be delivered (written, oral, digital, or on paper, internet, telephone, etc.), and when it should be delivered. As far as we know, this shorter assessment has not been validated against longer assessments. However, other studies measuring information needs among YACSs (see overview in Supplementary File; e.g., McCarthy et al. [11] and Christen et al. [10]) use such single-item questions to investigate the prevalence of specific information needs. A major strength of the study is its large nationwide, population-based sample of unselected long-term YACSs.

## Conclusion and clinical implications

Our study shows that a large proportion of YACSs report information needs regarding lifestyle and rehabilitation several years after treatment. Survivors diagnosed 5–10 years ago, who have undergone treatments other than minimal surgery, who have several late effects, in particular chronic fatigue and mental distress, and who are inactive and obese report higher needs than those without these conditions.

Health care personnel, both in a hospital setting and in general practice, who meet long-term YACSs should be prepared to provide lifestyle advice and information about them and refer to rehabilitation services if needed. Providing such information will meet the needs of many YACSs and hopefully enable them to make healthy lifestyle choices, potentially improving their long-term health.

## Supplementary Information

Below is the link to the electronic supplementary material.

Supplementary file1 (DOCX 17 KB)
