# Supplementary material for: Needs for information about lifestyle and rehabilitation in long-term young adult cancer survivors
Source: Support Care Cancer. 2021 Aug 1;30(1):521–33. doi: 10.1007/s00520-021-06418-z (PMC8636439; doi:10.1007/s00520-021-06418-z)
Supplement: Supplementary file 1 — Supplementary file1 (DOCX 17 KB) [file 520_2021_6418_MOESM1_ESM.docx]

### Supplementary table: Overview of studies information needs among (A)YACSs

| **Author**  **Year** | **N** | **Agegroup** | **Age at survey** | **Tid fra diagnose** | **Unmet needs** |
| --- | --- | --- | --- | --- | --- |
| Zebrack et al. 2007 | 1088 | YACS (15-35) | 18-39 | >3 år |  |
| Zebrack B. 2008 | 217 | YACS (15-35) | 18-40 |  | Information needs  Diet/nutrition: 89 % in need, 46 % unmet  Exercise: 86 % in need, 49 % unmet |
| Zebrack B. 2009 | 879 | YACS (15-35) | 18-39 | Mean 4.7 år | Information needs  Diet/nutrition: 80 % in need, 51 % unmet  Exercise: 79 % in need, 53 % unmet |
| Keegan et al. 2012 | 523 | AYACS (15-39)  (AYA HOPE) |  | median 11 months | Information:  >50% unmet info needs on cancer and treatment  40 % unmet info needs on nutrition and diet  32 % unmet info needs on staying physically fit or getting exercise  Services:  75 % unmet needs support groups  58 % unmet needs physical or occupational therapist for rehabilitation  56 % unmet needs psychiatrist, psychologist, social worker or mental health worker |
| Smith et al 2013 | 484 | AYACS  (AYA HOPE) | 15-39 | 6-14 months | Services:  36% unmet need for at least one service  Most common unmet needs:  16 % financial service  15 % mental health service  14 % support group service |
| Zebrack et al 2014 | 215 | AYACS | 15-39 | 16 months | Information  >50 % unmet exercise information  >50 % diet/nutrition information  Counseling  >45 % champs, retreats, social programs |
| DeRouen et al 2015 | 484 | AYACS | 15-39 | Median 11 months | Information  28% high level of information needs (9-13 unmet needs)  39% medium level of information needs(4-8 unmet needs)  33% low level of information needs (0-3 unmet needs) |
| Shay et al  2017 | 1395 | AYACS |  | 39 % >5 years | Information needs unmet  78 % Late-effects  45 % Fertility  Recurrence concerns  Family risk of cancer concerns |
| Christen et al. 2018 | 160 | AYACS (16-25) |  | >5år | Current Information needs  58 % disease  56 % treatment  71 % follow-up  79 % late-effects |
| MCCarthy et al 2018 | 196 | AYACS (15-25) |  | < 24 months | Information  40% unmet info needs on long-term effects  30 % unmet info needs on nutrition and diet  32 % unmet info needs on staying physically fit or getting exercise |
